# Long-term effectiveness and side-effects of intranasal esketamine in treatment-resistant depression: real-world, single-arm study of over 100 sessions

**DOI:** 10.1192/bjo.2025.10950

**Published:** 2026-01-23

**Authors:** Nawfel Ayad, Karim Abdel Aziz, Samer Makhoul, Ban Abbas, Syed Fahad Javaid

**Affiliations:** Al Reem Neuroscience Centre, Abu Dhabi, United Arab Emirates; Department of Psychiatry, College of Medicine and Health Sciences, United Arab Emirates University, Al-Ain, United Arab Emirates

**Keywords:** Treatment-resistant depression, esketamine, long-term effectiveness, real-world evidence, maintenance therapy

## Abstract

**Background:**

Treatment-resistant depression (TRD) poses a significant clinical challenge, with limited evidence guiding long-term pharmacological strategies. Esketamine, a glutamatergic modulator, has demonstrated short-term efficacy in TRD, but data on its extended use in real-world settings remains scarce.

**Aims:**

This study aimed to evaluate the long-term effectiveness and side-effects of intranasal esketamine in adults with TRD over more than 100 treatment sessions.

**Method:**

We conducted a retrospective, single-arm, pre–post study of 20 patients with TRD at a psychiatric out-patient clinic in the United Arab Emirates. All participants received ≥100 sessions of intranasal esketamine alongside oral antidepressants. Depression and anxiety symptoms were assessed with the Patient Health Questionnaire-9 (PHQ-9) and Generalised Anxiety Disorder-7 (GAD-7) scales. Side-effects were monitored through blood pressure, sedation, dissociation, urinary symptoms and psychiatric symptoms.

**Results:**

After an average of 129 esketamine sessions (mean duration 2.5 years), PHQ-9 and GAD-7 scores significantly decreased (*P* < 0.001). A total of 85% of patients improved in depressive severity, with 25% achieving remission; 65% improved in anxiety severity, and 20% reached remission. Esketamine was generally well tolerated; side-effects were mild and transient, with no serious adverse events. However, urinary symptoms suggestive of cystitis occurred in 20% of patients, highlighting the need for ongoing monitoring in long-term treatment.

**Conclusions:**

Intranasal esketamine demonstrated sustained effectiveness and an acceptable side-effect profile in a real-world TRD cohort with extensive psychiatric comorbidity. These findings support its long-term use in complex clinical populations, and underscore the need for further prospective, multi-site studies.

Major depressive disorder (MDD) is a common psychiatric disorder with a global lifetime prevalence ranging from 2 to over 20%,^1^ and is currently one of the leading causes of disability worldwide.^2^ It has a tendency to chronicity and a variable response to treatment,^3^ with more than a third of patients failing to fully respond to adequate doses and durations of antidepressants, resulting in residual symptoms, treatment unresponsiveness and impaired levels of social and occupational functioning.^4,5^ Treatment resistance is characterised by an absence of symptomatic remission after the use of two successive trials of antidepressants of different pharmacological classes, of adequate dose and duration while ensuring good adherence.^6,7^ A wide range of sociodemographic (e.g. female gender, age, financial insecurity, low level of education, etc.) and clinical factors (e.g. psychiatric and physical comorbidities) are associated with treatment resistance,^7^ and its tendency increases with the number of pharmacological trials.^8^ The Sequenced Treatment Alternatives to Relieve Depression (STAR*D) trial highlighted declining rates in both remission and response as patients progressed through each of the four successive treatment levels (36.8, 30.6, 13.7 and 13% for remission, and 48.6, 28.5, 16.8 and 16.3% for response).^9^ Therefore, it is important to consider strategies that can alleviate clinical symptoms as early as possible in the course of the illness. There are several approaches for managing treatment-resistant depression (TRD), including optimisation of the current antidepressant, switching to a different antidepressant, augmentation with an atypical antipsychotic or lithium, or combination with another antidepressant, psychotherapy or neurostimulation therapies such as electroconvulsive therapy, repetitive transcranial magnetic stimulation and vagus nerve stimulation.^10,11^

## Esketamine

Because some patients still do not respond to these approaches, additional therapeutic strategies are needed. In recent years, a growing body of research has supported the role of the glutamatergic system in the pathogenesis of depression, especially the role of N-methyl-D-aspartate (NMDA) receptors as a potential pharmacotherapeutic target for MDD, including TRD.^12^ Esketamine is the S-enantiomer of ketamine, a general anaesthetic, that works by blocking NMDA receptors, particularly on inhibitory gamma-aminobutyric acid-ergic interneurons, transiently enhancing glutamatergic activity. This increased glutamate activity then triggers a cascade of events, including the activation of α-amino-3-hydroxy-5-methyl-4-isoxazolepropionic acid receptors, the release of brain-derived neurotrophic factor and the activation of downstream signalling pathways. These processes ultimately lead to increased synaptic plasticity and improved synaptic connections in brain regions involved in mood regulation, resulting in reduction of depressive symptoms. Esketamine may also improve dysfunctional dopaminergic transmission, potentially helping reduce symptoms such as anhedonia.^13^

First introduced for medical use as an anaesthetic in 1997, esketamine was approved as an antidepressant by the US Food and Drug Administration and the European Medicines Agency in 2019, followed by approvals from more than 75 health authorities worldwide.^14^ A number of guidelines such as the Canadian Network for Mood and Anxiety Treatments and American Psychiatric Association endorse its adjunctive use in TRD after the failure of two or more antidepressant trials.^15,16^ Available as a nasal spray, esketamine delivers its dose via two sprays (one per nostril) and, in combination with an oral antidepressant, has demonstrated a statistically significant reduction in depressive symptoms in TRD compared with oral antidepressants and placebo.^17^ It has also demonstrated a sustained decreased risk of relapse among stable remitters and responders in long-term trials.^18^

Despite promising short-term efficacy, the long-term effectiveness, safety and tolerability of intranasal esketamine remain underexplored. Most published evidence is limited to durations under a year. The largest study with extensive long-term data is SUSTAIN-3, an open-label extension trial evaluating more than 1100 patients over a mean of 3.6 years, with many receiving well over 100 sessions of esketamine. It showed durable symptom reduction and no new safety concerns.^19^ However, SUSTAIN-3 represents data from a highly controlled trial environment, limiting its applicability to real-world settings. Other observational studies, such as the Asian subgroup analyses from SUSTAIN-2, describe safety and efficacy for up to a year.^20^ However, few, if any, published studies comprehensively track symptoms, tolerability and side-effects over more than 100 sessions in routine practice. In addition to its positive impact on clinical symptoms of depression, there is emerging evidence of esketamine’s role in improving the quality of life in individuals with TRD.^21,22^

Additionally, to date there are no published studies that report data from non-Western countries, further limiting the generalisability of existing long-term findings. The current study addresses this gap by investigating detailed outcomes from a Middle Eastern setting by analysing data for patients with TRD treated for over 100 esketamine sessions (almost 2 years), with specific monitoring of comorbidities, concurrent medications and adverse effects. This contributes uniquely by providing real-world, patient-level insights into the long-term use of esketamine in complex clinical populations. Therefore, we sought to investigate the long-term, real-world effectiveness of esketamine in TRD in a sample of patients with and without comorbid psychiatric disorders. We hypothesised that long-term intranasal esketamine treatment for more than 100 sessions would result in a significant reduction in depressive symptom severity in patients with TRD (as measured by the Patient Health Questionnaire-9 (PHQ-9)).

## Method

We conducted a retrospective, single-arm, pre–post study evaluating the effectiveness and side-effects of long-term intranasal esketamine in a sample of patients with TRD receiving more than 100 sessions, comparing clinical outcomes before and after treatment with esketamine. We collected and analysed data obtained from the computerised medical record system for patients between June 2021 and June 2025 who attended the Al Reem Neuroscience Centre, a private psychiatric service, in Abu Dhabi, United Arab Emirates.

### Participants

Our inclusion criteria were as follows: male and female patients aged 18–65 years, with a primary DSM-5 diagnosis of MDD, who had a PHQ-9 score of >5 when starting treatment with esketamine, had not responded to an adequate dose and duration (6–8 weeks) of at least two different classes of antidepressant drugs and had been receiving esketamine for >100 sessions. We excluded patients aged <18 years; those without a primary diagnosis of MDD; those with a PHQ-9 score of <5; those who had not had at least two adequate trials of different classes of antidepressants; those who received <100 sessions of intranasal esketamine; patients receiving concurrent non-pharmacological treatments for depression (such as psychotherapy, transcranial magnetic stimulation or electroconvulsive therapy), to ensure that symptom changes could be attributed primarily to esketamine rather than to these adjunctive interventions; and those with a history of any clinically significant or any uncontrolled medical conditions (e.g. hypertension, diabetes mellitus and neurological disorders).

The authors assert that all procedures contributing to this work comply with the ethical standards of the relevant national and institutional committees on human experimentation and with the Helsinki Declaration of 1975, as revised in 2013. All procedures involving human patients were approved by Alreem Hospital Research Ethics Committee (approval number: MF7272-2025-002). Patients attending the out-patient service and fulfilling criteria for TRD were offered the option of intranasal esketamine treatment for major depression. Before starting treatment, all patients were asked to sign a voluntary consent form stating that their data may be used anonymously for research purposes.

### Measures

Data were collected for patient demographic data (age, gender) and clinical data (primary psychiatric diagnosis, number and type of psychiatric comorbidities; current medication and dose for depression; previous number and type of medications for depression; number of esketamine sessions received; systolic and diastolic blood pressure before, 5 min after and 1 h after esketamine treatment; heart rate before, 5 min after and 1 h after treatment; oxygen saturation during treatment).

Data for assessment of symptoms was collected using the following measures.

#### **PHQ-9** ^23^

This is a brief self-administered questionnaire used to assess the severity of depression and to monitor the severity of symptoms over time. It consists of nine questions based on the DSM-IV diagnostic criteria for MDD. Each item is rated on a scale from 0 to 3 (0 = not at all, 1 = several days, 2 = more than half the days and 3 = nearly every day). Scores are added up to give a total score, ranging from 0 to 27, with severity of depression categorised as follows: 0–4: none or minimal symptoms, 5–9: mild depression, 10–14: moderate depression, 15–19: moderate to severe depression and 20–27: severe depression.

#### **GAD-7** ^24^

This is a widely used self-administered tool used to screen and assess the severity of generalised anxiety disorder. It consists of seven items (nervousness, inability to stop worrying, excessive worry, restlessness, difficulty in relaxing, easy irritation and fear of something awful happening). Each item is rated on a scale from 0 to 3 (0 = not at all, 1 = several days, 2 = more than half the days and 3 = nearly every day). Scores are added up to give a total score, ranging from 0 to 21, with severity of anxiety categorised as follows: 0–4: minimal anxiety, 5–9: mild anxiety, 10–14: moderate anxiety and 15–21: severe anxiety.

The following measures were administered to evaluate the long-term safety and side-effect profile associated with extended treatment. These measures were collected once at the end of treatment (after the final esketamine session), to capture cumulative or residual side-effects following prolonged exposure, rather than to assess acute or short-term effects over time.

#### **Brief Psychiatric Rating Scale (BPRS)** ^25^

This is an 18-item, clinician-rated scale that measures the severity and presence of various psychiatric symptoms, including anxiety, depression, hallucinations and disorientation. Each item is rated on a scale from 1 to 7 (1 = not present, 2 = very mild, 3 = mild, 4 = moderate, 5 = moderately severe, 6 = severe and 7 = extremely severe), to yield a total score between 18 and 126. We included the BPRS in our study to screen for psychiatric symptoms occurring as side-effects from esketamine.

#### **Modified Observer’s Assessment of Alertness and Sedation scale (MOAA/S)** ^26^

This scale was used to assess the level of alertness and sedation following esketamine treatment. Scores range from 0 to 5 (5 = responds readily to name spoken in normal tone, 4 = lethargic response to name spoken in normal tone, 3 = responds only after name is called loudly and/or repeatedly, 2 = responds only after mild prodding or shaking, 1 = responds only after painful trapezius squeeze, 0 = no response after painful trapezius squeeze). A score of 5 indicates the patient is awake or minimally sedated, whereas a score of 0 indicates a state of general anaesthesia or unresponsive to stimuli. Loss of response is typically defined as a MOAA/S score below 4, indicating moderate sedation. Deep sedation is often defined as MOAA/S scores of 1–2.

#### **Clinician-Administered Dissociative States Scale (CADSS):** ^27^

This is a 23-item clinician-administered scale used to assess dissociative symptoms in patients following esketamine treatment, and is the most frequently used measure of dissociation in clinical trials of ketamine and esketamine in TRD.^28^ Each item is rated on a scale from 0 to 4 (0 = not at all, 1 = mild, 2 = moderate, 3 = severe, 4 = extreme) with total scores ranging from 0 to 92 and higher scores indicating more severe dissociation. A cut-off score of 30 or above often indicates dissociative psychopathology. The CADSS was used to assess patients three times: once before esketamine treatment, 5 min after receiving the treatment and 1 h after receiving the treatment.

#### **Bladder Pain/Interstitial Cystitis Symptom Score (BPIC-SS):** ^29^

This is a self-administered 8-item scale that measures the severity of bladder pain/ interstitial cystitis in the past 7 days that may occur with esketamine treatment. There are seven items that are rated from 0 to 4 (0 = not at all, 1 = a little, 2 = somewhat, 3 = moderately, 4 = a great deal). There is one item for the ‘persistent urge to urinate’, two items for the ‘necessity to urinate driven by bladder pain’, two items for ‘bother associated with daytime and nighttime frequency’ and three items for ‘bladder pressure/pain’. The eighth item asks the subject to rate their bladder pain from 0 (no bladder pain) to 10 (worst bladder pain) in the past 7 days. Item scores are added up to give a total score ranging from 0 to 38, with higher scores indicating more severe symptoms.

### Esketamine procedure

Esketamine was prescribed as recommended by the US Food and Drug Administration.^30^ All participants received intranasal esketamine co-administered with an oral antidepressant, with each device delivering 28 mg via two sprays (one in each nostril). Before receiving esketamine, patients were asked to sign a consent form confirming their understanding of the details of the treatment, including benefits and risks, after these were explained to them. Patients who met criteria for and consented to receiving the treatment were administered esketamine under the supervision of a psychiatrist in an out-patient clinic setting. Because of the risk of nausea and vomiting, patients were advised to avoid food for at least 2 h before administration and to avoid drinking liquids at least 30 min before administration. Blood pressure (systolic and diastolic) and heart rate were assessed before, immediately after and 1 h after administration. Oxygen saturation was monitored for at least 2 h after receiving esketamine. The esketamine dose was initiated at 56 mg (two devices), and adjusted on an individual basis during the treatment period in accordance with the following recommendations: weeks 1–4: 56 or 84 mg twice weekly; weeks 5–8: 56 or 84 mg once weekly; and week 9 and onward: 56 or 84 mg once every 1 or 2 weeks.

### Statistical analysis

Data were recorded and analysed with SPSS for Windows, Version 29.0 (2022). We used mean and s.d. for parametric data, median and interquartile range (s.d.) for non-parametric data, and frequency and percentage for non-numerical (qualitative) data. We compared total and individual PHQ-9 and GAD-7 scores before and after treatment by using Wilcoxon signed-rank tests. To control for multiple comparisons, *P*-values were adjusted with the Benjamini–Hochberg false discovery rate procedure. Longitudinal PHQ-9 and GAD-7 scores were visualised with spaghetti plots to depict individual symptom trajectories across baseline, every 20 sessions and the final session. Each participant’s trajectory was plotted as a thin grey line, with the group median shown as a bold black line. A *P*-value of <0.05 was considered statistically significant.

## Results

We identified 20 patients who met the inclusion criteria for their data to be included in our analysis and were receiving esketamine for more than 100 sessions. All 20 patients were initiated on two devices (56 mg) in the first session, and then maintained on three devices (84 mg) from the second session onward. Eighteen patients (90%) continued on weekly sessions for the duration of their esketamine treatment, whereas two patients (10%) switched to sessions every 2 weeks, one after 127 sessions and one after 159 sessions.

### Demographic and clinical characteristics

Ten patients (50%) were male. The mean age for the sample was 40 years, ranging from 28 to 64 years. The mean duration of the depressive illness was 6.4 years (ranging from 2 to 25 years). Nineteen patients (95%) had a comorbid psychiatric diagnosis. The mean number of psychiatric comorbidities was three comorbidities, ranging from 0 to 6 comorbidities. Generalised anxiety disorder was the most frequent comorbid diagnosis (75%). Fluoxetine was the most frequently prescribed current antidepressant (40%), whereas venlafaxine was the most frequently prescribed previous medication for depression (65%). The mean number of previous antidepressant trials was three trials. The mean number of esketamine sessions received was 129, ranging from 100 to 202 sessions, with a mean duration of treatment of 127 weeks (ranging from 96 to 198 weeks), or 2.5 years (ranging from 1.9 to 3.8 years). Table 1 summarises the demographic and clinical characteristics of the sample.

**Table 1:** Demographic and clinical characteristics

Characteristic	Value
Age in years, mean (s.d.)	40.25 (8.747)
Gender, *n (*%)	
Male	10 (50)
Female	10 (50)
Duration of depressive illness in years, mean (s.d.)	6.40 (6.065)
Frequency of comorbidities, *n* (%)	
No comorbidities	1 (5)
One comorbidity	0
Two comorbidities	8 (40)
Three comorbidities	4 (20)
Four comorbidities	4 (20)
Five comorbidities	1 (5)
Six comorbidities	2 (10)
Type of comorbidities, *n (*%)	
GAD	15 (75)
Insomnia	13 (65)
PTSD	10 (50)
Panic disorder	5 (25)
Alcohol use disorder	5 (25)
Adult ADHD	4 (20)
OCD	3 (15)
Borderline personality disorder	3 (15)
Somatic symptom disorder	2 (10)
Social anxiety disorder	1 (5)
Number of comorbidities, mean (s.d.)	3.05 (1.504)
Number of esketamine sessions, mean (s.d.)	129.25 (31.571)
Duration of esketamine treatment in weeks, mean (s.d.)	126.70 (33.17)
Duration of esketamine treatment in years, mean (s.d.)	2.45 (0.6345)
Current concomitant medications and doses, *n* (%)	
Bupropion (150–450 mg)	3 (15)
Agomelatine (25 mg)	1 (5))
Escitalopram (20 mg)	2 (10)
Quetiapine (50–200 mg)	3 (15)
Venlafaxine (150–300 mg)	2 (10)
Aripiprazole (5 mg)	2 (10)
Fluoxetine (40–80 mg)	8 (40)
Cariprazine (1.5–3 mg)	4 (20)
Clomipramine (300 mg)	1 (5)
Mirtazapine (30 mg)	1 (5)
Lamotrigine (200 mg)	1 (5)
Sertraline (100–150 mg)	2 (10)
Desvenlafaxine (400 mg)	1 (5)
Olanzapine (5 mg)	1 (5)
Lurasidone (40 mg)	1 (5)
Vortioxetine (10–30 mg)	2 (10)
Paroxetine (60 mg)	1 (5)
Lisdexamphetamine (60–70 mg)	2 (10)
Brexpiprazole (1–2 mg)	2 (10)
Previous antidepressant trials, mean (s.d.)	3.35 (2.852)
Previous medications for depression, *n* (%)	
Sertraline	10 (50)
Venlafaxine	13 (65)
Quetiapine	8 (40)
Olanzapine	4 (20)
Lurasidone	8 (40)
Cariprazine	8 (40)
Brexpiprazole	8 (40)
Mirtazapine	6 (30)
Vortioxetine	6 (30)
Duloxetine	3 (15)
Aripiprazole	1 (5)
Lamotrigine	6 (30)
Agomelatine	3 (15)
Clomipramine	4 (20)
Risperidone	2 (10)
Desvenlafaxine	5 (25)
Paroxetine	5 (25)
Bupropion	3 (15)
Fluoxetine	1 (5)
Paliperidone	1 (5)
Amitriptyline	2 (10)
Escitalopram	7 (35)

GAD, generalised anxiety disorder; PTSD, post-traumatic stress disorder; ADHD, attention-deficit hyperactivity disorder; OCD, obsessive–compulsive disorder.

### PHQ-9 and GAD-7 scores and categories

**Fig. 1:**
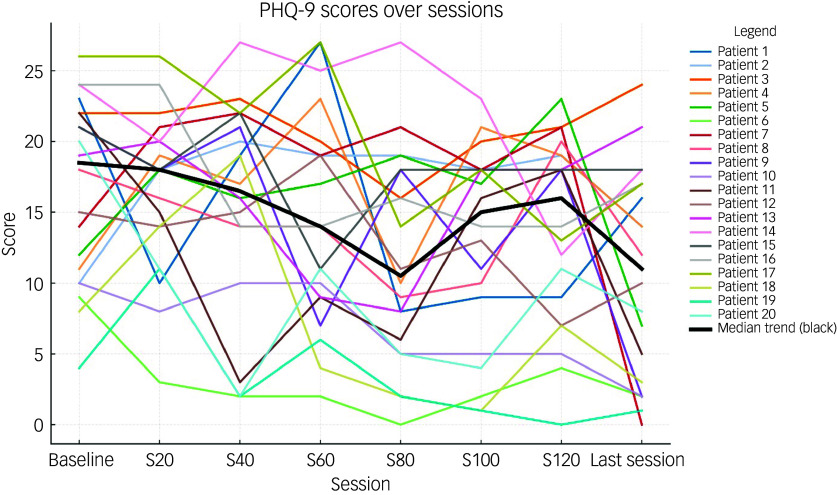
Individual PHQ-9 trajectories across treatment sessions (baseline, 20, 40, 60, 80, 100, 120 and last session). Each line represents one participant; the bold black line represents the group median. PHQ-9, Patient Health Questionnaire-9.

**Fig. 2:**
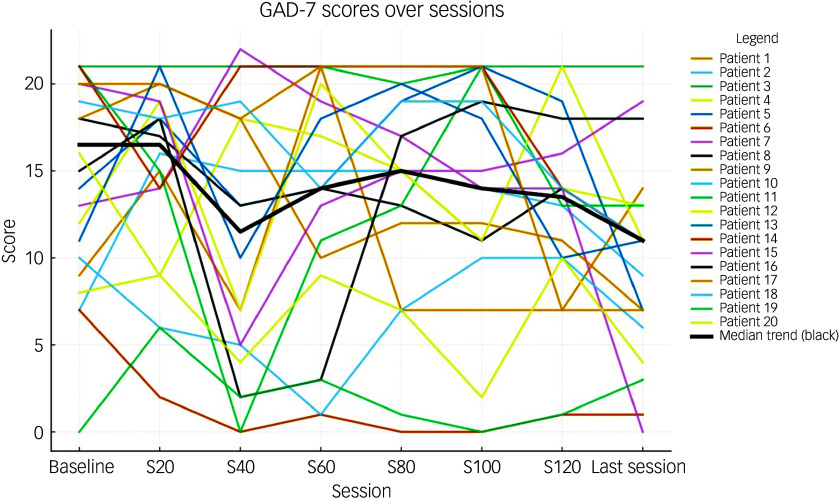
Individual GAD-7 trajectories across treatment sessions (baseline, 20, 40, 60, 80, 100, 120 and last session). Each line represents one participant; the bold black line represents the group median. GAD-7, Generalised Anxiety Disorder-7.

Table 2 shows the total and individual scores on the PHQ-9 and GAD-7, the categories of severity and change in severity before receiving and after the last session of esketamine. Wilcoxon signed-rank tests showed a statistically significant improvement (*P* < 0.05) in both PHQ-9 and GAD-7 total scores between before receiving the first dose and after receiving the last dose of esketamine, indicating a significant improvement of both depressive and anxiety symptoms (Table 2). There were statistically significant improvements in six out of the nine symptoms on the PHQ-9, and six out of the seven symptoms on the GAD-7 (Table 2). Seventeen patients (85%) experienced a clinical improvement in the severity of depressive symptoms on the PHQ-9, of whom five (25%) achieved remission after the last esketamine session (PHQ-9 score <5). Thirteen patients (65%) experienced a clinical improvement in the severity of anxiety symptoms on the GAD-7, of whom four (20%) achieved remission after the last esketamine session (GAD-7 score <5). Two patients experienced mild anxiety following the last esketamine session, after previously reporting minimal anxiety before starting esketamine (Table 2). Figures 1 and 2 display individual PHQ-9 and GAD-7 trajectories across the full treatment course. The plots reveal an overall downward trend in depressive and anxiety symptoms, with the greatest improvement seen within the first 40–60 sessions and maintained thereafter.

**Table 2:** PHQ-9 and GAD-7 scores and categories

PHQ-9 and GAD-7 scores	Median [IQR] before first esketamine session	Median [IQR] after last esketamine session	Difference (median)	Wilcoxon *W*	*P*-value
PHQ-9 total score	18.50 [13.38–23.62]	11.00 [6.12–15.88]	7.5	13.5	0.0002^*^
Loss of interest/pleasure	2.00 [1.00–3.00]	1.00 [0.38–1.62]	1.0	12.0	0.004^*^
Depressed mood	2.50 [1.50–3.50]	1.00 [0.38–1.62]	1.5	15.5	0.01^*^
Sleep disturbances	2.00 [1.00–3.00]	2.00 [1.38–2.62]	0.0	42.0	0.801
Fatigue or loss of energy	1.50 [0.50–2.50]	1.50 [1.00–2.00]	0.0	12.0	0.1898
Change in appetite	2.00 [1.00–3.00]	1.50 [0.88–2.12]	0.5	24.0	0.06
Feelings of worthlessness or guilt	3.00 [2.00–4.00]	1.00 [0.00–2.00]	2.0	6.0	<0.001^*^
Difficulty concentrating	2.00 [1.38–2.62]	1.00 [0.00–2.00]	1.0	12.0	0.004^*^
Psychomotor agitation or retardation	1.00 [0.38–1.62]	0.50 [−0.50 to 1.50]	0.5	16.5	0.02^*^
Suicidal thoughts	2.00 [0.50–3.50]	0.00 [−0.12 to 0.12]	2.0	7.0	0.004^*^
GAD-7 total score	16.50 [11.75–21.25]	10.50 [6.62–14.38]	6.0	16.5	0.001^*^
Feeling nervous, anxious or on edge	2.00 [1.50–2.50]	1.00 [0.50–1.50]	1.0	12.0	0.001^*^
Difficulty controlling anxiety/worry	2.00 [1.50–2.50]	1.00 [0.50–1.50]	1.0	5.5	0.001^*^
Excessive worry about multiple topics	2.50 [2.00–3.00]	1.00 [0.50–1.50]	1.5	5.0	0.002^*^
Difficulty relaxing	2.00 [1.00–3.00]	2.00 [1.50–2.50]	0.0	24.0	0.1062
Physical restlessness and fidgeting	2.00 [1.00–3.00]	1.50 [0.50–2.50]	0.5	18.0	0.02^*^
Easily annoyed/irritable	2.00 [1.00–3.00]	1.00 [0.50–1.50]	1.0	26.0	0.02^*^
Fear of negative outcomes	2.00 [1.00–3.00]	1.00 [0.38–1.62]	1.0	36.5	0.045^*^

PHQ-9 category, *n* (%)	Before first esketamine session	After last esketamine session	GAD-7 category, *n* (%)	Before first esketamine session	After last esketamine session
Mild (5–9)	3 (15)	3 (15)	Minimal (0–4)	1 (5)	5 (25)
Moderate (10–14)	4 (20)	7 (35)	Mild (5–9)	4 (20)	4 (20)
Moderately severe (15–19)	4 (20)	4 (20)	Moderate (10–14)	4 (20)	9 (45)
Severe (20–27)	9 (45)	1 (5)	Severe (15–21)	11 (55)	2 (10)
Improved severity	–	17 (85)	Improved severity	–	13 (65)
Remission achieved	–	5 (25)	Remission achieved	–	4 (20)
Unchanged severity	–	3 (15)	Unchanged severity	–	5 (25)
Worsening severity	–	0	Worsening severity	–	2 (10)

PHQ-9, Patient Health Questionnaire-9; GAD-7, Generalised Anxiety Disorder-7; IQR, interquartile range.

Wilcoxon signed-rank tests were used for all pre–post comparisons. *P*-values were adjusted for multiple comparisons with the Benjamini–Hochberg false discovery rate.

* Statistically significant (*P* < 0.05).

### Side-effects of esketamine

All side-effect measures (CADSS, MOAA/S, BPIC-SS and BPRS) were administered once, after the final treatment session, to evaluate cumulative or residual side-effects following long-term esketamine use. One patient experienced a slight increase in blood pressure (reaching 150/92 mmHg) after receiving esketamine (Table 3). No patients experienced a significant increase in heart rate or experienced a significant decrease in oxygen saturation after receiving esketamine (Table 3). A total of three patients (15%) reported experiencing mild dissociative symptoms (CADSS scores: 34–41) 5 mins after receiving the third device of esketamine, all resolving within 1 h (CADSS score <30) (Table 3). Eight patients (40%) reported experiencing mild sedation (MOAA/S score: 4) (Table 3). Four patients (20%) reported experiencing urinary symptoms (positive on the BPIC-SS): one patient reported being bothered by frequent urination during the daytime and by having to get up during the night to urinate; one patient reported urinating to avoid pain in their bladder from getting worse; one patient reported urinating because of pain in their bladder, and had a feeling of pressure in their bladder; and one patient reported still feeling the need to urinate just after they had urinated (Table 3). Baseline BPIC-SS data were not available for comparison, and no follow-up urinalysis or urological assessment was documented for these patients. Consequently, the temporal relationship between esketamine exposure and urinary symptoms could not be determined. The mean BPRS score was 24, ranging from 18 to 39. Sixteen patients (80%) reported experiencing at least one symptom on the BPRS. The most frequently reported symptom on the BPRS was excitement (45%) (Table 3).

**Table 3:** Side-effects of esketamine

Side-effect	Value
Mean CADSS score before final esketamine session (s.d.)	0
Mean CADSS score 5 min after third device of final esketamine session (s.d.)	14.10 (12.460)
Mean CADSS score 1 h after third device of final esketamine session (s.d.)	4.35 (6.262)
CADSS-positive 5 min after third device of final esketamine session, *n* (%)	3 (15)
CADSS-positive 1 h after third device of final esketamine session, *n* (%)	0
Mean MOAA/S (s.d.)	4.60 (0.503)
MOAA/S category, *n* (%)	
Score of 5	12 (60)
Score of 4	8 (40)
Mean BPIC-SS total score (s.d.)	0.50 (1.235)
BPIC-SS-positive, *n* (%)	4 (20)
Frequency of BPIC-SS symptoms, *n* (%)	
Bothered by frequent urination during daytime	1 (5)
Bothered by getting up during the night to urinate	1 (5)
Urinates to avoid pain in bladder getting worse	1 (5)
Urinated because of pain in bladder	1 (5)
Had a feeling of pressure in their bladder	1 (5)
Felt the need to urinate just after having urinated	1 (5)
Patients with elevated blood pressure after receiving esketamine, *n* (%)^a^	1 (5)
Patients with elevated heart rate after receiving esketamine, *n* (%)^b^	0 (0)
Patients with decreased oxygen saturation after receiving esketamine, *n* (%)^c^	0 (0)
BPRS-positive, *n* (%)	16 (80)
Mean total BPRS score (s.d.)	23.90 (6.155)
Frequency of BPRS symptoms, *n* (%)	
Somatic concern	4 (20)
Anxiety	1 (5)
Emotional withdrawal	1 (5)
Conceptual disorganisation	1 (5)
Guilt feelings	0
Tension	2 (10)
Mannerisms and posturing	0
Grandiosity	5 (25)
Depressive mood	0
Hostility	1 (5)
Suspiciousness	0
Hallucinatory behaviour	1 (5)
Motor retardation	5 (25)
Uncooperativeness	1 (5)
Unusual thought content	3 (15)
Blunted affect	2 (10)
Excitement	9 (45)
Disorientation	1 (5)

CADSS, Clinician-Administered Dissociative States Scale; MOAA/S, Modified Observer’s Assessment of Alertness and Sedation scale; BPIC-SS, Bladder Pain/Interstitial Cystitis Symptom Score; BPRS, Brief Psychiatric Rating Scale.

a. Elevated blood pressure defined as systolic ≥140 mmHg or diastolic ≥90 mmHg.

b. Elevated heart rate defined as >100 beats per minute.

c. Decreased oxygen saturation defined as <95%.

## Discussion

This retrospective, real-world study examined the long-term effectiveness and side-effects of intranasal esketamine in patients with TRD who received over 100 treatment sessions across an average of 2.5 years. The results demonstrate a statistically and clinically significant reduction in depressive and anxiety symptoms, as measured by PHQ-9 and GAD-7 scores, respectively. Notably, 85% of participants showed improvement in depression severity, and 25% achieved remission. Similarly, 65% improved in anxiety severity, with 20% achieving remission. These findings contribute to the growing evidence base supporting esketamine’s long-term efficacy, particularly in real-world settings. Although previous studies such as SUSTAIN-2 and SUSTAIN-3 have demonstrated durability of symptom reduction with intranasal esketamine,^14,18^ these were largely conducted in highly controlled trial environments. Our study adds to this literature by offering insight from a naturalistic clinical practice in a Middle Eastern setting, expanding the generalisability of esketamine’s long-term outcomes across diverse populations. The inclusion of patients with multiple psychiatric comorbidities (95% of the sample) and high antidepressant burden (mean of three prior trials) also highlights its utility in complex, difficult-to-treat populations often excluded from controlled trials.

Our study observed significant improvements in core depressive symptoms, including anhedonia, depressed mood, worthlessness, concentration difficulties, psychomotor changes and suicidality. These findings are consistent with previous studies showing esketamine’s rapid and robust effects on mood, including suicidality and anhedonia.^17,31,32^ However, other studies have reported less consistent benefits across individual symptom domains, such as psychomotor symptoms. Sobreiro et al^33^ found that although esketamine was effective in improving processing speed for 3 months, improvement was not sustained, with performance actually decreasing at 6 months.

Our study also demonstrated improvements in comorbid anxiety symptoms reducing GAD-7 scores by a median of 6 points, which was similar to other reports, such as Fedgchin et al,^34^ who also reported that esketamine reduced GAD-7 scores in patients with TRD, with a mean score reduction of 7.4 on 56 mg and 7.7 on 84 mg. The longitudinal visualisations further confirm that symptom improvement was both rapid and durable, supporting the long-term effectiveness of esketamine treatment observed in this cohort.

In terms of safety, esketamine was generally well-tolerated in our cohort. Side-effects were mild and transient, with no serious adverse events reported such as severe or prolonged dissociation after sessions, suicidal ideation during or after sessions, or persistent delusions or manic symptoms after long-term use of esketamine. These findings are consistent with controlled trials.^18,20^ However, concerns about long-term safety and abuse potential remain, especially regarding urological side-effects with chronic use, especially in patients with high cumulative doses.^19^ Approximately a fifth of participants reported urinary symptoms suggestive of cystitis during treatment. Although we cannot confirm whether these were pre-existing, treatment-emergent or persistent, this finding raises the possibility of a cystitis-related safety signal in long-term esketamine use. Given the established association between chronic ketamine exposure and urological complications,^35^ larger and prospective studies with standardised urological evaluation are warranted to better characterise this risk.

Although exploring predictors of treatment response would have been valuable, our small sample size precluded reliable multivariable analysis without a high risk of statistical overfitting. Therefore, we did not include a regression analysis. A particularly interesting question is whether the intensity of dissociative symptoms, as measured by the CADSS, predicts antidepressant response to esketamine. Future studies with larger samples are needed to address this important question, and to identify other clinical or biological predictors of response to long-term esketamine treatment.

### Limitations

There were several limitations to this study. First, the retrospective, uncontrolled design limits causal inference. Second, the modest sample size (*N* = 20) reduces power for detecting subtle effects and may limit generalisability. Third, all patients were drawn from a single centre in the United Arab Emirates, potentially introducing selection bias. Fourth, symptom improvement was based on self-report measures, which, although validated, are subject to reporting bias. The PHQ-9 and GAD-7, although widely used and validated for routine clinical assessment, are primarily screening instruments. The PHQ-9 in particular is less sensitive to change than clinician-rated scales such as the Montgomery–Åsberg Depression Rating Scale, and does not capture the full range of depressive symptom dimensions. This may have limited the precision of our measurement of symptom improvement. We also did not measure functional or quality-of-life outcomes, which may be crucial for evaluating real-world impact. Furthermore, as only patients who completed at least 100 sessions were included, our sample inherently consisted of individuals who tolerated esketamine well. In addition, the study cannot fully evaluate treatment tolerability or discontinuation rates. Also, side-effect measures (CADSS, MOAA/S, BPIC-SS and BPRS) were administered once after the final session, which limits the ability to evaluate how these symptoms may have changed during the course of treatment. Another limitation was the exclusion of patients receiving concurrent non-pharmacological treatments (psychotherapy, transcranial magnetic stimulation or electroconvulsive therapy), which may limit generalisability to broader clinical settings where such combined approaches are common.

### Implications

Despite these limitations and the variability noted across studies, our findings provide valuable real-world evidence that intranasal esketamine can be an effective long-term treatment option for TRD in complex, comorbid populations. The sustained improvements in depression and anxiety symptoms over more than 100 sessions suggest that esketamine may be a viable maintenance strategy in patients who are unresponsive to traditional treatments.

However, the generally limited evidence for longer-term effects and side-effects of esketamine calls for further prospective research. Future investigations should focus on identifying reliable predictors of response, evaluating functional and cognitive outcomes, and assessing long-term safety over extended treatment durations. Large-scale, multi-centre studies, including diverse populations, will be essential to refine patient selection and optimise long-term treatment strategies with esketamine. Given the emerging evidence linking chronic ketamine exposure with cystitis and other urological complications,^35^ our findings underscore the importance of systematic monitoring for urological side-effects during prolonged esketamine therapy.

## Data Availability

The data that support the findings of this case are available from the corresponding author, K.A.A., upon reasonable request.

## References

[1] Gutiérrez-Rojas L , Porras-Segovia A , Dunne H , Andrade-González N , Cervilla JA. Prevalence and correlates of major depressive disorder: a systematic review. Rev Bras Psiquiatr 2020; 42: 657–72. 32756809 10.1590/1516-4446-2019-0650 PMC7678895

[2] Rong J , Wang X , Cheng P , Li D , Zhao D. Global, regional and national burden of depressive disorders and attributable risk factors, 1990-2021: results from the 2021 Global Burden of Disease study. Br J Psychiatry 2025; 227: 688–97. 39809717 10.1192/bjp.2024.266

[3] Cleare A , Pariante CM , Young AH , Anderson IM , Christmas D , Cowen PJ , et al. Evidence-based guidelines for treating depressive disorders with antidepressants: a revision of the 2008 British Association for Psychopharmacology guidelines. J Psychopharmacol 2015; 29: 459–525. 25969470 10.1177/0269881115581093

[4] Al-Harbi KS. Treatment-resistant depression: therapeutic trends, challenges, and future directions. Patient Prefer Adherence 2012; 6: 369–88. 22654508 10.2147/PPA.S29716 PMC3363299

[5] Fava M , Davidson KG. Definition and epidemiology of treatment-resistant depression. Psychiatr Clin North Am 1996; 19: 179–200. 8827185 10.1016/s0193-953x(05)70283-5

[6] Holtzmann J , Richieri R , Saba G , Allaïli N , Bation R , Moliere F , et al. Quelle définition pour la dépression résistante? [How to define treatment-resistant depression?]. Presse Med 2016; 45: 323–8. 26970938 10.1016/j.lpm.2016.02.002

[7] Rush AJ , Thase ME , Dubé S. Research issues in the study of difficult-to-treat depression. Biol Psychiatry 2003; 53: 743–53. 12706958 10.1016/s0006-3223(03)00088-x

[8] Warden D , Rush AJ , Trivedi MH , Fava M , Wisniewski SR. The STAR*D project results: a comprehensive review of findings. Curr Psychiatry Rep 2007; 9: 449–59. 18221624 10.1007/s11920-007-0061-3

[9] Sinyor M , Schaffer A , Levitt A. The Sequenced Treatment Alternatives to Relieve Depression (STAR*D) trial: a review. Can J Psychiatry 2010; 55: 126–35. 20370962 10.1177/070674371005500303

[10] McAllister-Williams RH , Arango C , Blier P , Demyttenaere K , Falkai P , Gorwood P , et al. The identification, assessment and management of difficult-to-treat depression: an international consensus statement. J Affect Disord 2020; 267: 264–82. 32217227 10.1016/j.jad.2020.02.023

[11] Rush AJ , Trivedi MH , Wisniewski SR , Nierenberg AA , Stewart JW , Warden D , et al. Acute and longer-term outcomes in depressed outpatients requiring one or several treatment steps: a STAR*D report. Am J Psychiatry 2006; 163: 1905–17. 17074942 10.1176/ajp.2006.163.11.1905

[12] Kadriu B , Musazzi L , Henter ID , Graves M , Popoli M , Zarate CA. Glutamatergic neurotransmission: pathway to developing novel rapid-acting antidepressant treatments. Int J Neuropsychopharmacol 2019; 22: 119–35. 30445512 10.1093/ijnp/pyy094 PMC6368372

[13] Kawczak P , Feszak I , Bączek T. Ketamine, esketamine, and arketamine: their mechanisms of action and applications in the treatment of depression and alleviation of depressive symptoms. Biomedicines 2024; 12: 2283. 39457596 10.3390/biomedicines12102283 PMC11505277

[14] Zaki N , Chen LN , Lane R , Doherty T , Drevets WC , Morrison RL , et al. Safety and efficacy with esketamine in treatment-resistant depression: long-term extension study. Int J Neuropsychopharmacol 2025; 28: pyaf027. 40319349 10.1093/ijnp/pyaf027 PMC12143125

[15] Lam RW , Kennedy SH , Adams C , Bahji A , Beaulieu S , Bhat V , et al. Canadian Network for Mood and Anxiety Treatments (CANMAT) 2023 update on clinical guidelines for management of major depressive disorder in adults. Can J Psychiatry 2024; 69: 641–87. 38711351 10.1177/07067437241245384 PMC11351064

[16] American Psychiatric Association. Practice Guideline for the Treatment of Patients with Major Depressive Disorder 3rd ed. APA, 2020.

[17] Popova V , Daly EJ , Trivedi M , Cooper K , Lane R , Lim P , et al. Efficacy and safety of flexibly dosed esketamine nasal spray combined with a newly initiated oral antidepressant in treatment-resistant depression: a randomized double-blind active-controlled study. Am J Psychiatry 2019; 176: 428–38. 31109201 10.1176/appi.ajp.2019.19020172

[18] Wajs E , Aluisio L , Holder R , Daly EJ , Lane R , Lim P , et al. Esketamine nasal spray plus oral antidepressant in patients with treatment-resistant depression: assessment of long-term safety in a phase 3, open-label study (SUSTAIN-2). J Clin Psychiatry 2020; 81: 19m12891. 10.4088/JCP.19m12891 32316080

[19] Zaki N , Chen LN , Lane R , Doherty T , Drevets WC , Morrison RL , et al. Long-term safety and maintenance of response with esketamine nasal spray in participants with treatment-resistant depression: interim results of the SUSTAIN-3 study. Neuropsychopharmacology 2023; 48: 1225–33. 37173512 10.1038/s41386-023-01577-5 PMC10267177

[20] Jeon HJ , Ju PC , Sulaiman AH , Aziz SA , Paik JW , Tan W , et al. Long-term safety and efficacy of esketamine nasal spray plus an oral antidepressant in patients with treatment-resistant depression: an Asian sub-group analysis from the SUSTAIN-2 study. Clin Psychopharmacol Neurosci 2022; 20: 70–86. 35078950 10.9758/cpn.2022.20.1.70 PMC8813327

[21] Cheng MCH , Dri CE , Ballum H , Valentino K , Cheung W , Teopiz KM , et al. The effects of ketamine and esketamine on measures of quality of life in major depressive disorder and treatment-resistant depression: a systematic review. J Affect Disord 2025; 382: 438–42. 40274121 10.1016/j.jad.2025.04.119

[22] Jamieson C , Popova V , Daly E , Cooper K , Drevets WC , Rozjabek HM , et al. Assessment of health-related quality of life and health status in patients with treatment-resistant depression treated with esketamine nasal spray plus an oral antidepressant. Health Qual Life Outcomes 2023; 21: 40. 37158911 10.1186/s12955-023-02113-1 PMC10169482

[23] Kroenke K , Spitzer RL , Williams JB. The PHQ-9: validity of a brief depression severity measure. J Gen Intern Med 2001; 16: 606–13. 11556941 10.1046/j.1525-1497.2001.016009606.x PMC1495268

[24] Spitzer RL , Kroenke K , Williams JB , Löwe B. A brief measure for assessing generalized anxiety disorder: the GAD-7. Arch Intern Med 2006; 166: 1092–7. 16717171 10.1001/archinte.166.10.1092

[25] Overall JE , Gorham DR. The brief psychiatric rating scale. Psychol Rep 1962; 10: 799–812.

[26] Chernik DA , Gillings D , Laine H , Hendler J , Silver JM , Davidson AB , et al. Validity and reliability of the Observers Assessment of Alertness/Sedation Scale: study with intravenous midazolam. J Clin Psychopharmacol 1990; 10: 244–51. 2286697

[27] Bremner JD , Krystal JH , Putnam FW , Southwick SM , Marmar C , Charney DS , et al. Measurement of dissociative states with the Clinician-Administered Dissociative States Scale (CADSS). J Trauma Stress 1998; 11: 125–36. 9479681 10.1023/A:1024465317902

[28] Bremner JD , Williamson D , Vaccarino V. Psychometric properties of the 23-Item Clinician Administered Dissociative States Scale (CADSS) in a psychological trauma population. J Affect Disord 2024; 364: 249–58. 39147159 10.1016/j.jad.2024.08.050 PMC11365742

[29] Humphrey L , Arbuckle R , Moldwin R , Nordling J , van de Merwe JP , Meunier J , et al. The bladder pain/interstitial cystitis symptom score: development, validation, and identification of a cut score. Eur Urol 2012; 61: 271–9. 22050826 10.1016/j.eururo.2011.10.004

[30] US Food and Drug Administration. Spravato (Esketamine) Label. FDA, 2025 (https://www.accessdata.fda.gov/drugsatfda_docs/label/2025/211243s016lbl.pdf [cited 10 Jun 2025]).

[31] Daly EJ , Trivedi MH , Janik A , Li H , Zhang Y , Li X , et al. Efficacy of esketamine nasal spray plus oral antidepressant treatment for relapse prevention in patients with treatment-resistant depression: a randomized clinical trial. JAMA Psychiatry 2019; 76: 893–903. 31166571 10.1001/jamapsychiatry.2019.1189 PMC6551577

[32] Canuso CM , Singh JB , Fedgchin M , Alphs L , Lane R , Lim P , et al. Efficacy and safety of intranasal esketamine for the rapid reduction of symptoms of depression and suicidality in patients at imminent risk for suicide: results of a double-blind, randomized, placebo-controlled study. Am J Psychiatry 2018; 175: 620–30. 29656663 10.1176/appi.ajp.2018.17060720

[33] Sobreiro MFM , Silveira PSP , Cavenaghi VB , da Costa LP , de Souza BPF , Takahashi RES , et al. Long-term cognitive outcomes of esketamine nasal spray in treatment-resistant depression: a preliminary report. Pharmaceuticals (Basel) 2025; 18: 173. 40005986 10.3390/ph18020173 PMC11858642

[34] Fedgchin M , Trivedi M , Daly EJ , Melkote R , Lane R , Lim P , et al. Efficacy and safety of fixed-dose esketamine nasal spray combined with a new oral antidepressant in treatment-resistant depression: results of a randomized, double-blind, active-controlled study (TRANSFORM-1). Int J Neuropsychopharmacol 2019; 22: 616–30. 31290965 10.1093/ijnp/pyz039 PMC6822141

[35] Anderson DJ , Zhou J , Cao D , McDonald M , Guenther M , Hasoon J , et al. Ketamine-induced cystitis: a comprehensive review of the urologic effects of this psychoactive drug. Health Psychol Res 2022; 10: 38247. 36118982 10.52965/001c.38247 PMC9476224

